# Crowd-based requirements elicitation via pull feedback: method and case studies

**DOI:** 10.1007/s00766-022-00384-6

**Published:** 2022-08-20

**Authors:** Jelle Wouters, Abel Menkveld, Sjaak Brinkkemper, Fabiano Dalpiaz

**Affiliations:** 1 Royal Netherlands Marechaussee, The Hague, The Netherlands; 2 Tournify, Amsterdam, The Netherlands; 3 Utrecht University, Utrecht, The Netherlands

**Keywords:** CrowdRE, Elicitation, User stories, Case studies, Pull feedback

## Abstract

Crowd-based Requirements Engineering (CrowdRE) promotes the active involvement of a large number of stakeholders in RE activities. A prominent strand of CrowdRE research concerns the creation and use of online platforms for a crowd of stakeholders to formulate ideas, which serve as an additional input for requirements elicitation. Most of the reported case studies are of small size, and they analyze the size of the crowd, rather than the quality of the collected ideas. By means of an iterative design that includes three case studies conducted at two organizations, we present the CREUS method for crowd-based elicitation via user stories. Besides reporting the details of these case studies and quantitative results on the number of participants, ideas, votes, etc., a key contribution of this paper is a qualitative analysis of the elicited ideas. To analyze the quality of the user stories, we apply criteria from the Quality User Story framework, we calculate automated text readability metrics, and we check for the presence of vague words. We also study whether the user stories can be linked to software qualities, and the specificity of the ideas. Based on the results, we distill six key findings regarding CREUS and, more generally, for CrowdRE via pull feedback.

## Introduction

Crowd-based Requirements Engineering (CrowdRE) is an emerging paradigm for Requirements Engineering (RE) that promotes the active involvement of a “crowd” of stakeholders, including the current and potential users, of a software product [1]. CrowdRE expands the reach of established RE approaches [2], which involve a selected sample of the stakeholders, extending the notion of market-driven RE [3, 4] toward the democratic participation of users in RE [5].

So far, CrowdRE research has mainly investigated requirements elicitation [6]: “the process of seeking, uncovering, acquiring, and elaborating requirements for computer-based systems” [7]. CrowdRE researchers [1] have proposed two approaches for complementing existing elicitation techniques with requirements-related feedback from the users^1^: (i) in *pull feedback*, the crowd is requested to express their needs and wishes through a dedicated feedback channel; and (ii) in *push feedback*, the users initiate the process of providing feedback, e.g., by sending feedback through an app store.

Our research focuses on crowd-based elicitation via *pull feedback*; we study the acquisition of feedback in the form of user stories [8] through an web platform. We report on three case studies within two organizations. The $\textit{Tournify}$ case regards a tournament management app that is developed by a namesake software start-up company. The *V-Sys* and *S-Sys* cases concern information systems at the Royal Netherlands Marechaussee (RNLN or, based on the Dutch name, KMar), part of the Ministry of Defence of the Netherlands.

We tackle two limitations of existing research. First, thanks to our collaboration with non-academic organizations, we report on cases with a larger *size* (number of users, ideas, and votes) than existing attempts to apply CrowdRE in practice [2, 9, 10]. Second, we go beyond the quantitative assessment of CrowdRE by conducting a *qualitative analysis* of the crowd-generated ideas.

In this paper, we make the following contributions:

- Through an iterative design process that involves three case studies, we propose the *CREUS* method: *Crowd-based Requirements Elicitation with User Stories*. *CREUS* supports the conduction of pull-based elicitation of requirements via an online platform. We present a precise characterization of *CREUS* by means of the Process-Deliverable-Diagram notation [11].
- We analyze the results from the three case studies in a time-boxed, experimental period in which different versions of the *CREUS* method were used to ask a crowd to provide feedback using an online feedback channel. These conducted case studies are among the largest in size, to date.
- We qualitatively analyze the ideas from the experimental period and beyond, where possible, in terms of whether they are high-quality user stories, their vagueness, if they can be linked to quality requirements, the text readability, and the generality or specificity of the ideas.

This paper builds on and consolidates our previous work. The *REfine* platform [9] was our first systematic approach for crowd-based elicitation via pull feedback. In that research, we could gather only a limited crowd though. The *CREUS* method is the outcome of an iterative design process that relies on the conducted case studies reported earlier: $\textit{Tournify}$ [12], *S-Sys* and *V-Sys* [13]. The qualitative analysis of the ideas is a novel contribution of this paper.

We use the term *idea* to refer to the crowd inputs. In *CREUS*, ideas are gathered using the user story notation [8, 14]: As a $\langle$role$\rangle$, I want to $\langle$action$\rangle$, so that $\langle$benefit$\rangle$. While some of these ideas may be directly mapped to a *requirement*, other ideas need further discussion and refinement by the analysts prior to becoming requirements.

*Organization*. In Sect. 2, we present the relevant background and the related work. Section 3 presents our research method, while Sect. 4 describes the *CREUS* method through a Process-Deliverable Diagram. The main results from the case studies are discussed in Sect. 5. The collected ideas are analyzed qualitatively in Sect. 6. We presents the key findings and draw conclusions in Sect. 7. Finally, we discuss limitations and future directions in Sect. 8.

## Background and related work

We first introduce the elementary background on CrowdRE in Sect. 2.1. Then, we discuss previous CrowdRE via elicitation platforms in Sect. 2.2. Finally, we review alternative approaches for conducting CrowdRE in Sect. 2.3.

### Background on CrowdRE

CrowdRE is defined by Groen et al. as an “umbrella term for all automated RE techniques, including crowdsourcing, text mining and data mining” [15] that can be utilized to actively involve a crowd of stakeholders, including users, in the RE process. Their proposed approach encompasses multiple methods: both quantitative data (using mining techniques) and qualitative feedback (using crowdsourcing) are collected as a source of requirements.

Independently, other research groups conducted studies along the same lines. Snijders et al. introduced the term *Crowd-centric requirements engineering* [16]. They justified CrowdRE saying that “users are seldom involved, despite the common agreement that doing it would result in better requirements elicitation and higher chances for project success” [16]. Similarly, Johann and Maalej [5] offered a perspective in favor of the democratic participation of masses of users in the RE process, which they called Liquid RE.

Hosseini et al. [17] also studied crowdsourcing in requirements elicitation. Due to the fast-changing landscape of IT products, especially with the introduction of software-as-a-service and cloud products, they argued that the user groups of these products would become more heterogeneous. Therefore, established requirements elicitation efforts might not be effective, but using crowdsourcing to gather requirements might be.

Many of these researchers co-authored a landscape paper [1] that distinguishes between two main approaches to CrowdRE: (i) *pull feedback* concerns the provision of a feedback channel for the crowd to formulate their ideas; and (ii) *push feedback* denotes user-initiated feedback processes, e.g., through the authoring of reviews in an app store. Both streams of user requirements are then analyzed by a product team in order to further improve the software system at hand. The same paper [1] also compares CrowdRE to market-driven RE [4], explaining that CrowdRE can be seen as a “logical upscale form of market-driven RE”, the same way market-driven RE enables “customer-specific RE to transcend the organization’s boundaries”. Furthermore, as per the cases at the KMar in this paper, CrowdRE can also be applied to cases where the software product is not released on the market.

### CrowdRE via elicitation platforms

One of the earliest crowd-based elicitation platforms, developed before the term CrowdRE was coined, was the *Requirements Bazaar* by Renzel et al. [10]. This web-based platform supports requirements elicitation by providing tools for co-creation and prioritization. With *Requirements Bazaar*, users are able to formulate ideas and to prioritize them. Several projects were and are being conducted using this platform, although the results are not reported in depth.

Fernandes et al.’s *iThink* [18] is a game-based collaborative tool for idea generation. The introduction of game elements aims at heightening the engagement of the participants. The case studies with iThink [19] are, however, limited to small groups of users. The *REfine* platform by Snijders et al. [9], together with its supporting crowd-based RE method [16], combines the gamification aspects of iThink with the aim of engaging a crowd of users for an internal software product. In a case study concerning the internal users of a product, REfine led to 21 needs, 37 comments and 130 votes, which were provided by 19 active crowd members. The participants indicated that they were more engaged than in different requirements elicitation efforts. However, this study is of limited size and shows the difficulty of engaging a large crowd.

The *GARUSO* platform [2] was built with the aim of involving stakeholders that are outside organizational reach. GARUSO went beyond REfine in terms of gamification, by offering a game-like experience to the participants, which was expected to engage them for a longer time. The researchers managed to involve 32 active stakeholders (from the 700+ participants who visited the platform), and they contributed 56 ideas.

In 2019, Glinz gave an overview of the status and future of CrowdRE [20]. Glinz remarks that the existing case studies have a limited size (see also Table 2), and he also points out challenges such as the high-number of features that only few users want, overlooking minorities, and sustaining user motivation. This paper makes steps forward by reporting on case studies with larger crowd sizes and with a detailed analysis of the CrowdRE inputs.

### Alternative approaches within CrowdRE

Feedback channels have been explored by numerous scholars. The most frequently investigated channel consists of reviews in app stores [21–24], which allows the users of mobile apps to express their feedback without the necessity to provide an ad-hoc elicitation platform. According to Maalej and colleagues, the feedback in this kind of channel may contain a variety of requirements-relevant information such as feature requests, bug reports, and praises [22]. Panichella et al. [23] propose a more refined classification scheme that includes feature requests, opinion asking, problem discovery, solution proposal, information seeking, and information giving.

The research in the app store analysis is vast and goes well beyond the scope of this paper [25]. Among the most relevant works in the RE domain, we mention the extraction of reviews that concern a particular feature [26]; the classification of user reviews among categories such as bugs and feature requests [21–23]; and the analysis of the reviews’ sentiment [27]. Researchers have also studied the use of user reviews for comparing apps in the same category: the RE-SWOT technique [24] applies the well-known Strength-Weakness-Opportunity-Threat (SWOT) analysis [28] to compare the reviews implemented by one app producer to its competitors based on the user rating; Garousi et al. [29] analyze COVID-19 tracing apps with the objective of identifying similarities and differences in the user reviews.

A demographic study [30] of user engagement in app stores shows some difficulties with this feedback channel, for users (i) are mostly review readers rather than review authors, (ii) find it easier to switch to a competing app instead of providing feedback, and (iii) perceive that resolving their issues would take too long. While interesting, this channel is only applicable to apps that are made available publicly on an app store.

Researchers have also studied other feedback channels such as Twitter [31, 32] or online fora [33–35]. The major difficulty regarding Twitter is that requirements-relevant information is scattered within a sheer amount of interactions that take place on such a broad channel. Online discussions in user forums are closer to our research, as they may be seen as a more structured way of expressing and discussing the collected ideas. Future research should consider this feedback platform and compare it to the inputs obtained via the type of elicitation platforms that are discussed in this paper.

Another CrowdRE approach is the use of a crowd-work platform, where crowd workers are paid for the execution of RE-related tasks. This technique has been studied in the context of generating creative ideas [36], classifying app reviews according to software product qualities [37], and extracting requirements from privacy policies [38]. In our work, however, we focus on collecting and analyzing the feedback that is provided by the users of a system, rather than on the involvement of additional, external crowd workers.

## Research method

We are interested in studying the feasibility and effectiveness of crowd-based requirements elicitation via pull feedback as a tool to enable the users of software systems to express ideas. In particular, we define two research questions, each leading to a phase of our research:

1. *RQ1. What method can support requirements engineers in the adoption of crowd-based elicitation via pull feedback?* This research question is set with a practical use case in mind, that of assisting the practitioners who may want to use crowd-based elicitation but do not have access to methods that are tested in practice. To address RQ1, we follow Wieringa’s design science research methodology [39] and conduct three case studies. Each case study is an iteration of the so-called *design cycle*: we investigate the problem at a host organization, we design a solution that consists of an evolved version of our elicitation method, and we validate the solution in that organizational context. The outcomes of each iteration (summarized in Sect. 5) feed the following one, and the final result is the *CREUS* method described in Sect. 4.
2. *RQ2. What types of ideas are prevalent when deploying crowd-based elicitation methods via pull feedback?* After the completion of the three case studies, we conduct a qualitative, empirical analysis of the collected ideas aimed at characterizing the ideas according to multiple classification schema. The aim of this second phase of this research, reported in Sect. 6, is to provide researchers and practitioners with a detailed analysis of the types of ideas, so to better understand how this elicitation method can complement other ones.

Note that we explore the role of crowd-based elicitation *in addition to* established elicitation methods, not as a replacement. Besides its function for gathering new ideas and for assessing the perceived importance assigned by the users [21], user involvement has been shown to increase system usage and acceptance [40] as well as system success [41].

- *Iterative design of the CREUS method via case studies (RQ1)*

In the first research step, we answer RQ1 through the iterative design of the *CREUS* method via multiple iterations of Wieringa’s design cycle, one for each of the case studies listed in the introduction: $\textit{Tournify}$, *S-Sys*, and *V-Sys*. In each iteration, we employ crowd-based elicitation to address one case-specific goal (see Sect. 5.1), and the obtained results contribute to evolving *CREUS*.

Each case study is conducted by following the principles of Canonical Action Research (CAR), “one of the more widely practiced and reported forms of action research in the Information Systems literature” [42].

The CAR principle of *change through action research* was employed in all cases: crowd-based requirements elicitation was used in projects where the organizations had sub-optimal involvement of the users in their requirements engineering processes, and we applied crowd-based elicitation as the means to improve this situation. The CAR principle of *cyclical process model* was employed at the KMar by having the results from the first case (*S-Sys*) inform the planning and execution of the second case (*V-Sys*).

The first and second authors of this paper were acting as researcher-employee in the involved organizations (the first author at KMar, the second author at Tournify), while the other authors acted as supervisors; this aligns with the CAR principles of *collaboration between researcher and client*.

The effectiveness of this research phase is measured in terms of two aspects:

1. *Crowd-based elicitation analysis*: number of participants, of ideas, of comments, of votes, dynamics of participation over time, types of users.
2. *Ideas usefulness*: innovation, completeness for development, granularity, estimated workload.

Each case uses a subset of these indicators, depending on their relevance, practical constraints regarding their collection, and the usefulness of assessing them for the goals of the case study.

The outcome of this phase is the *CREUS* method that is presented in this paper. For better readability, we present the activities and artifacts of *CREUS* in Sect. 4 before discussing the results from the case studies in Sect. 5. For each case study, we explain in Sect. 5.1 how the employed version of the method differed from the final one presented in Sect. 4.

- *A-posteriori qualitative analysis of the elicited ideas (RQ2)*

In the second research phase, to address RQ2, we conduct a qualitative analysis of the ideas that were generated by the crowd. We consider all the ideas that were collected in the three cases: $\textit{Tournify}$, *S-Sys*, and *V-Sys*. For $\textit{Tournify}$, we also examine additional ideas that were posted after the case study period related to RQ1. We analyze each of the ideas by considering the aspects and metrics in Table 1.

**Table 1:** Metrics used for analyzing the ideas in the a-posteriori analysis

Metric	Description
User story quality [8]	
Well-formed	A user story includes at least a $\langle$role$\rangle$ and an $\langle$action$\rangle$
Atomic	A user story expresses a requirement for exactly one user-visible feature
Conceptually sound	The $\langle$action$\rangle$ expresses a feature and the $\langle$benefit$\rangle$ expresses a rationale
Problem-oriented	A user story only specifies the problem, not the solution to it
Vagueness	
Vagueness	Does the user story include one of the weak words from QUARS++ [43]? Is the occurrence of that word leading to a vague requirement?
Text readability	
Automated readability index	Complexity of a text in terms of average number of characters per words, and the average number of words per sentence [44]
Flesch reading-ease test	Text complexity in terms of the average number of word per sentence and the average number of syllables per word [45]
Quality requirements (ISO/IEC 25010 standard [46])	
Reliability	Degree to which a system, product or component performs specified functions under specified conditions for a specified period of time
Performance (efficiency)	Performance relative to the amount of resources used under stated conditions
Security	Degree to which a product or system protects information and data so that persons or other products or systems have the degree of data access appropriate to their types and levels of authorization
Compatibility	Degree to which a product, system or component can exchange information with other products, systems or components, and/or perform its required functions, while sharing the same hardware or software environment
Usability	Degree to which a product or system can be used by specified users to achieve specified goals with effectiveness, efficiency and satisfaction in a specified context of use
Generality versus Specificity	
General	The idea refers to the general user of the system, without limitation on certain usage contexts
Specific	The idea concerns specific user types or specific usage contexts

While the first three aspects (user story quality, vagueness, text readability) measure mostly the linguistic quality of the formulated ideas, the last two aspects (quality requirements, generality vs. specificity) concern the type of requirements that originate from the crowd members.

To maximize the reliability of the results, for user story quality and for the quality requirements, two researchers tagged the user stories independently, the inter-rater agreement was calculated, and then the disagreements were resolved via discussion rounds.

## Crowd-based requirements elicitation via the *CREUS* method

Based on our previous experience in crowd-based requirements elicitation [9], and following the iterative design process described in Sect. 3 that builds on the case studies with $\textit{Tournify}$ [12], *V-Sys* and *S-Sys* [13], we derive a general method, called *CREUS*: *CRowd-based Elicitation via User Stories*. *CREUS* can be used by practitioners or researchers who wishes to conduct such an elicitation activity that can complement other elicitation techniques.

**Fig. 1:**
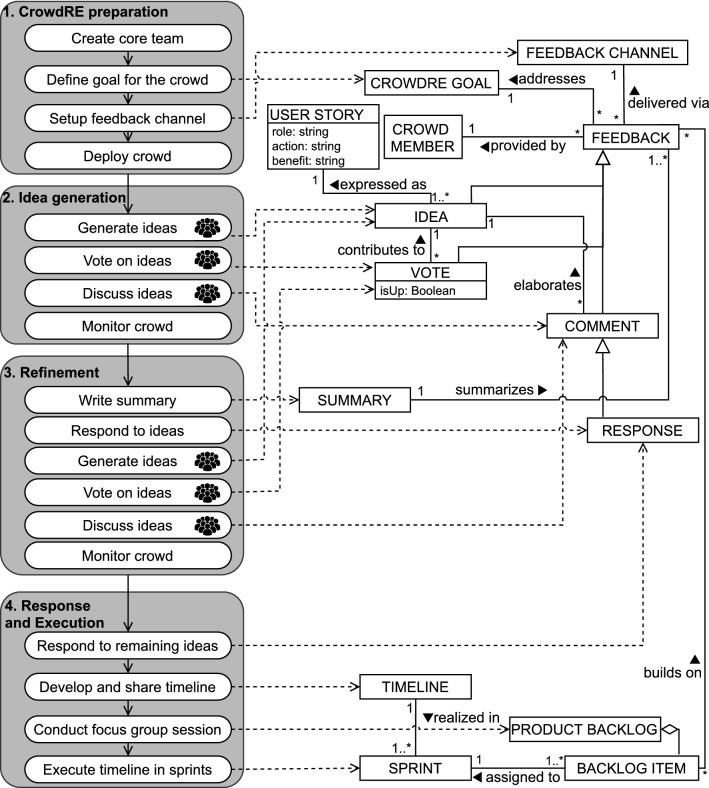
Process-Deliverable Diagram representing the *CREUS* method for crowd-based requirements elicitation. The activities with the  symbol are executed by the crowd, the others are performed by the core team

We present a precise description of *CREUS* using the Process-Deliverable Diagram (PDD) notation [11], which illustrates the activities and artifacts of a process. The PDD diagram is presented in Fig. 1, while the concept and the activity tables are in Appendix A in Table 13 and Table 14, respectively.

The *CREUS* method consists of four phases: *CrowdRE preparation*, *idea generation*, *refinement*, and *response and execution*. Three roles are active: the *core team* that coordinates the effort, the *crowd member* who contributes with feedback, and the *focus group member*, a crowd member who joins the discussions on how to implement the selected feedback.

While the four phases are linked sequentially in the PDD to show the conceptual steps of *CREUS*, it is possible to either (i) use *CREUS* in an agile manner by implementing ideas without waiting for the collection period to end, or (ii) leave the feedback channel open after an iteration of *CREUS*. The PDD is a guideline, not a prescriptive tool.

1. *CrowdRE preparation* A core team is created, which consists of requirements analysts who will oversee and manage the crowd. It is indeed important [9] to direct, motivate and sustain the crowd engagement. The core team first defines a goal for the crowd, which determines the primary aim of the crowd-based elicitation and that allows focused interaction through a feedback channel. The next step is the selection and configuration of the feedback channel to employ. This can range from general-purpose, commercial tools for idea generation (e.g., UserVoice or GetSatisfaction) to specific CrowdRE platforms [2, 9, 10]. Then, the core team advertises the channel and its purpose by inviting the prospective participants to join, thereby allowing crowd members to express their feedback.
2. *Idea generation* The invited crowd members can express their feedback via ideas, comments, and votes. In *CREUS*, ideas are formulated as user stories, as this notation allows to concisely state not only *what* the idea concerns, but also *who* would reap the benefit, and *why* this idea is important. Comments can be added to ideas to clarify vague ideas, to introduce possible variants or examples, and to offer counterpoints. Comments also enable the core team to ask for clarification to the crowd, if necessary. Finally, up/down-voting aims to estimate the degree to which an idea is shared among the crowd members. Throughout this second phase, the core team monitors the activity of the crowd and provides stimuli whenever necessary (e.g., by sending reminders to inactive crowd members).
3. *Refinement* While phase 2 focuses on idea divergence, phase 3 focuses on convergence thinking [47]: the existing ideas are consolidated to determine which ones to consider for implementation. The phase starts with the core team writing a summary of the ideas collected so far. This activity is especially useful for newcomers to obtain an overview of the existing feedback without browsing through all ideas in the feedback channel. Moreover, the core team writes responses to the ideas, so to highlight that their ideas are taken into account. The crowd is still able to generate, vote, and discuss ideas, as the responses of the core team might lead to new discussion points. Crowd monitoring activities continues like in phase 2.
4. *Response and execution* This phase denotes the transition from elicitation to the following phases of software development. First, the core team responds to not-yet-answered ideas. Second, a timeline is developed that describes to the team and to the crowd the time horizon for the development. Then, highly-engaged crowd members are invited by the core team to join a focus group that will prioritize the feedback, leading to the definition of a product backlog that consists of backlog items. A part of these backlog items build on the feedback provided by the crowd, while others originate from other elicitation activities as well as from the roadmap and long-term release planning of the product [4]. Finally, the timeline is executed in sprints, each of which is assigned a number of backlog items taken from the product backlog.

Note that, although the activities in phase 2 and 3 are unordered, votes and comments can only be posted for existing ideas. We do not prescribe a duration for phases 2 and 3. However, we can identify two general scenarios: (i) a time-bounded, activity-intense scenario in which the crowd focuses on a specific aspect of the system for a few weeks (e.g., enhancing the usability on mobile devices [9]); and (ii) a longer-term deployment in which the feedback channel is kept active for a longer time without restricting the scope (e.g., collecting inputs to improve the product [12]).

## Results from the case studies of crowd-based elicitation (RQ1)

We present the results from the three case studies with *CREUS*: the first phase of our research, which addresses RQ1. We present the goals of each of the case studies and the specifics of the elicitation method in Sect. 5.1. Then, we describe the feedback channels we employed in Sect. 5.2. After providing a quantitative overview of the outcomes in Sect. 5.3, we summarize the main results in Sect. 5.4 ($\textit{Tournify}$), Sect. 5.5 (*S-Sys*), and Sect. 5.6 (*V-Sys*). Extensive details regarding these case studies can be found in our previous work: for $\textit{Tournify}$, see Menkveld et al. [12]; for *S-Sys* and *V-Sys*, see Wouters et al. [13].

### Goals and details on the use of *CREUS*

In the first case, *CREUS* is used in the context of product evolution for an app, with the goal of *assessing the ability and eagerness of users to provide feedback in terms of user stories by means of an online platform*. The $\textit{Tournify}$ case concerns reaching out to the external users of the app, who would provide their inputs on a completely voluntary basis. *CREUS* was instantiated in an agile development process: the low-hanging fruit ideas were implemented before the end of the 5-week collection period (phases 2 and 3 in Fig. 1). The goal for the crowd was general: the company looked for ideas that would improve the current functionality as well as introduce new functions. Finally, the feedback channel was kept alive after the case study period and users continued using it.

In the second case, *CREUS* is used to elicit ideas for *S-Sys*, an operational system that will replace a legacy system. *S-Sys* will allow reporting on violations and offenses, and to generate formal police reports. A set of requirements were collected earlier using interviews, task analysis, and introspection. The main goal of this case study is to *validate whether* *CREUS* *will lead to similar requirements to those that were already gathered*. This study focuses on a single operational unit (“brigade”) within the KMar organization: the 478 employees of that brigade were invited to participate. *CREUS* was used to complement the existing requirements; the system would then be implemented by a external contractor after a tender process. As such, we focused on the first three phases of *CREUS*. Also, leaderboards were used (see Sect. 5.2) as a game element to foster user involvement.

In the third case, *CREUS* is used to identify ideas for a software product for which no requirements existed. The *V-Sys* product is going to replace another, outdated product at the KMar. The main goal is to *assess whether* *CREUS* *can be scaled up to the whole organization and, while doing so, is still able to produce useful ideas for the analysts who will have to specify the requirements for the system to-be*. *CREUS* was employed in a similar way as in the *S-Sys* case: the leaderboard was included as well, and the identified ideas would feed into a more comprehensive elicitation process. One key difference is that (see Sect. 5.6) the participants were invited to join in multiple rounds, due to practical constraints.

The KMar case studies targeted *operational employees*: the daily users of the systems for which requirements needed to be gathered. At the KMar, these employees are normally hardly involved in this process, even though they are the most important user group: their daily duties (e.g., police and border control tasks at airports) take priority over their participation in workshops and other requirements elicitation sessions.

### Feedback channels

The case studies reported in this paper are executed through the use of two purpose-made CrowdRE platforms, one per each involved organization.

For the $\textit{Tournify}$ case, the feedback platform was embedded in the website of the company, so that all users could access it.

**Fig. 2:**
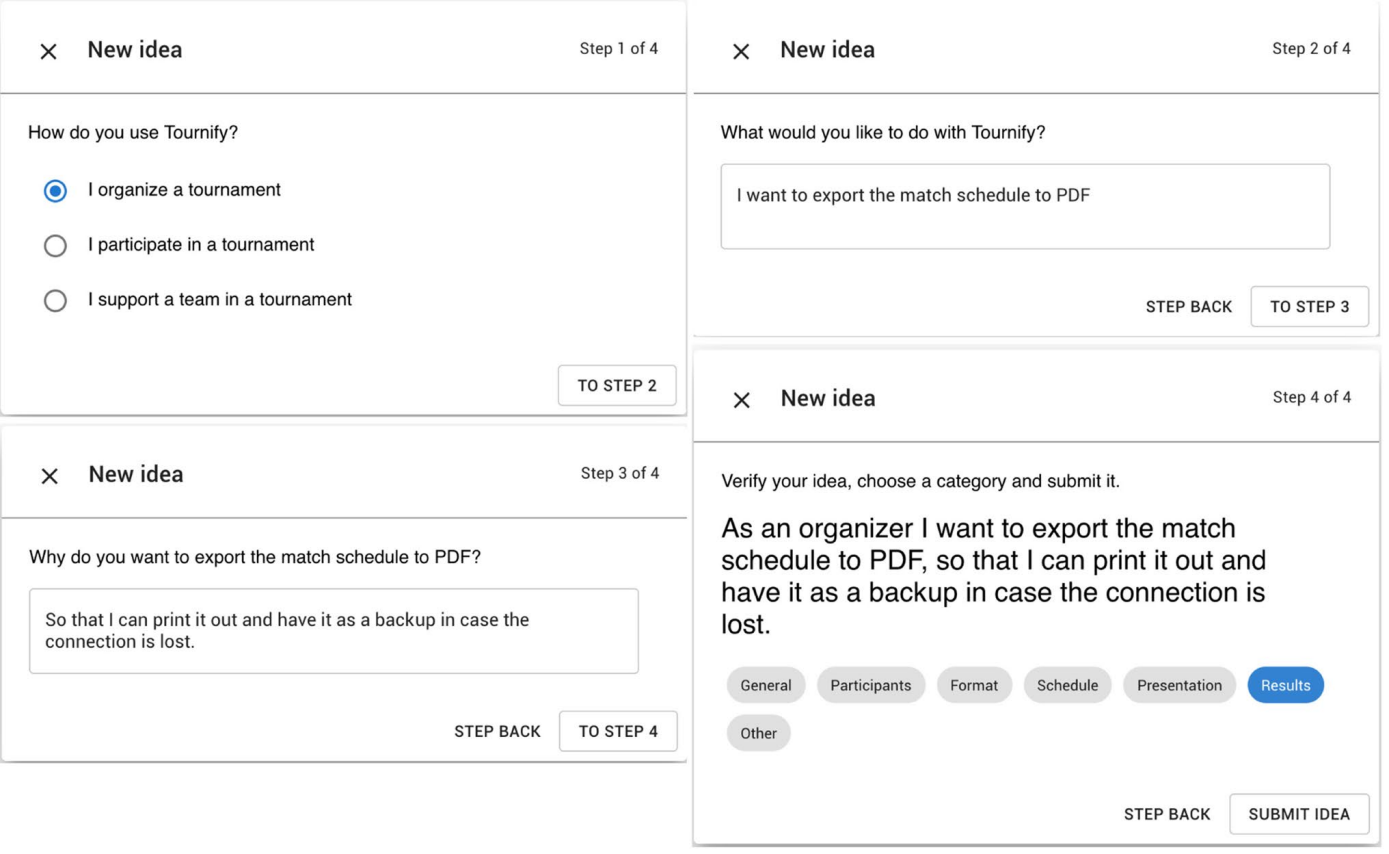
The wizard template for authoring user stories in the $\textit{Tournify}$ case

In order to help users formulate user stories, even if they have never used the notation before, we provided a wizard with four simple steps (Fig. 2): (i) the *role* is chosen among predefined options: organizer, participant, and supporter; (ii) the *goal* asks the user what s/he wants to do with $\textit{Tournify}$ via a textbox that contains the static text ‘I want to’ before the user input; (iii) the *benefit*, also requested via free text, which starts with ‘so that’; and (iv) *verification and category selection*: before submitting the idea, the user can verify the user story that has been assembled from the inputs, and they are asked to select one of the predefined categories, representing parts of the main menu of the application.

All requests are published on a grid visualization on the $\textit{Tournify}$ website, which can be accessed via the support menu. In addition to idea posting, the platform enables the other actions of phases 2 and 3 of *CREUS*: voting, commenting, and responding to the posted ideas.

**Fig. 3:**
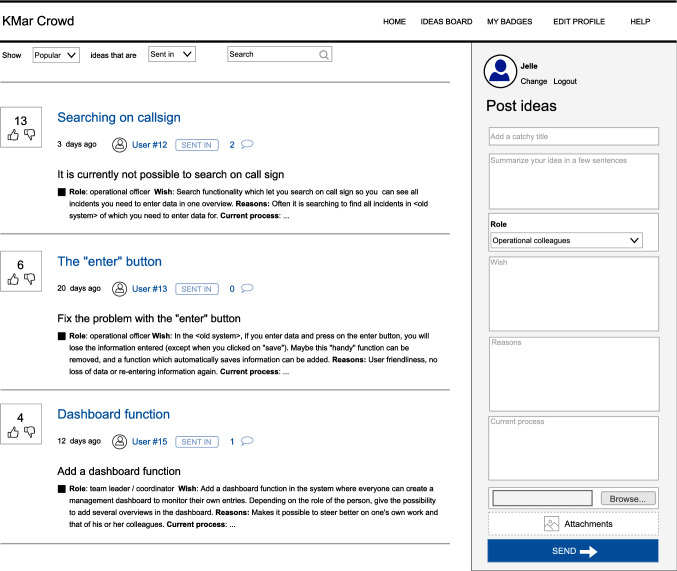
The idea board of the KMar Crowd platform, with data from *S-Sys* translated to English. On the left, the existing ideas together with voting buttons button are visible. On the right, new ideas can be expressed via a simplified user-story format

For *S-Sys* and *V-Sys*, the KMar Crowd platform was built by the first author on top of a WordPress site. The platform (illustrated in Fig. 3) supports phases 2 (idea generation) and 3 (refinement) of the *CREUS* method. Therefore, it allows participants to express user stories via a simplified format, and it allows voting and commenting. Furthermore, inspired by earlier research [9], it includes gamification elements: points, badges, and a leaderboard. The platform incorporates Single-Sign-On, which makes it possible to retrieve the origin of participants. When users open *CREUS* for the first time, they are asked to either fill in their real name or specify a pseudonym.

As users enter ideas, add comments, and up-/down-vote existing ideas, they gain points. When a certain amount of points of each category (ideas, comments, votes) is collected, users are rewarded with stars. As a positive reinforcement, all users start with one star after logging in for the first time. In the *S-Sys* and *V-Sys* case studies, all the participants who collected two or more stars were eligible for a small prize that was assigned via a raffle.

### Quantitative comparison of the outcomes

We relate our case studies to earlier empirical research with CrowdRE elicitation platforms. Table 2 summarizes the data from the three cases and contrasts them to the results from REfine [9] and GARUSO [2], the other studies which measured the quantity of feedback obtained via a dedicated platform. The table also includes column $\textit{Tournify}$ $^*$, which reports figures that include the outcomes obtained after the $\textit{Tournify}$ case study was concluded: the platform was left active and the users could provide their ideas for over two years.

**Table 2:** Comparison of the $\textit{Tournify}$, *S-Sys*, and *V-Sys* cases with earlier studies

Measurement	Tournify	S-Sys	V-Sys	Tournify$^*$	REfine	GARUSO
Duration in days	35	33	56	$\sim\,$ 1000	35	92
Participants						
Invited	337	478	2,393	unk.	37	unk.
Accessed	157	135	385	unk.	19	726
Active	39	60	130	$^\dag$135	19	32
Ideas	57	32	78	248	21	56
Logins	247	240	623	unk.	unk.	unk.
Votes	89	$^\ddag$284	$^\ddag$453	513	130	160
Comments	14	28	78	161	37	unk.
Ideas/Accessed	0.36	0.24	0.20	unk.	1.11	0.08
Ideas/Active	1.46	0.53	0.60	1.84	1.11	1.75

The $\textit{Tournify}$ $^*$ column refers to the additional ideas obtained from the channel after the case study period ended. $\dag$: for $\textit{Tournify}$ $^*$, we count only participants who posted ideas; for technical reasons, we could not record participants who voted or commented. $^\ddag$: these numbers are slightly lower than those reported in previous work [13], as every idea included a vote self-assigned to the author; for consistency, we subtracted those in this paper

We present three participant counts: *invited* is the number of people (possibly unknown) that were reached by an invitation to join the platform; *accessed* counts who visited the platform at least once; and *active* considers participants who interacted actively, by posting an idea, adding a comment, or expressing a vote. For the active participants of $\textit{Tournify}$ $^*$, we could only count those who posted at least one idea because of the information that was stored after the case study period (after the 35 days of $\textit{Tournify}$). The data regarding $\textit{Tournify}$ $^*$ is only used in Sect. 6 to answer RQ2.

Participant invitation differs per case study. For *S-Sys* and *V-Sys*, we used mass emails sent to the organization and physical briefings executed by team leaders. For $\textit{Tournify}$, the invitation to participate was included in the product for which ideas were gathered. For REfine, specific individuals were reached by the researchers, while GARUSO used targeted advertising to recruit participants through organizational mailing lists.

In addition to the participants’ counts, we present the number of ideas, of logins, of votes, comments, and the average number of ideas per participant who accessed and who was active on the platform.

The raw numbers provide a high-level overview, which will be enriched by the case-specific details in the following sections. We can see that our three cases had the highest number of *active* participants within the cases reported in the literature: 39, 60, and 130 for $\textit{Tournify}$, *S-Sys*, and *V-Sys*, respectively. The total number of votes for *S-Sys* and *V-Sys* is also high. When we look at the number of ideas per user who accessed or who was active, we see how the *S-Sys* and *V-Sys* cases lead to lower engagement than the $\textit{Tournify}$, REfine, or GARUSO. This may be justified by a few reasons: (i) the type of ideas that were formulated: see the analysis in Sect. 6; (ii) the organizational culture: KMar employees are used to conveying their inputs in a single, extensive message; and (iii) group dynamics: research has shown [48] that larger groups deliver a lower average of ideas per participant.

### Tournify

The feedback elicitation via a dedicated channel was announced via e-mail to 337 users who had shown some earlier participation by requesting a feature via another channel, by subscribing to the newsletter, or by making a purchase recently. A reminder was sent one month later. The data collection period was five weeks. One free $\textit{Tournify}$ upgrade was raffled among all active participants. Some ideas were accepted and were assigned the label *in development*, visible in the platform. Phase 4 of *CREUS* started while the ideas were being collected: one idea was implemented before the end of the elicitation period.

In the five-weeks period, 157 unique visitors accessed the platform. 39 of these users interacted with the platform by submitting an idea (23), voting (28), and/or commenting (nine). The active participants submitted a total of 57 ideas, 89 votes, and 14 comments (Table 2). The downvote idea functionality was never used. 65% (15) of the requesters submitted only one idea, two users submitted respectively two and three ideas, four users submitted five or more ideas, with a maximum of 14 ideas.

In 52% of the cases, the category assigned by the requester did not match the category assignment that the researcher-author would have assigned. This probably happened because we provided no guidance on the labels.

After the study, 13 active users responded to a questionnaire. Most of them (10) requested a feature, while the other three respondents only voted for a feature. They perceived the platform as very useful; their ratings on a 1-to-5 five-point Likert-type scale: posting ideas ($\overline{x} = 4.9$; $\sigma = 0.28$), viewing ($\overline{x} = 4.8$; $\sigma = 0.38$), voting ($\overline{x} = 4.5$; $\sigma = 0.88$), and commenting ($\overline{x} = 4.5$; $\sigma = 0.66$).

**Fig. 4:**
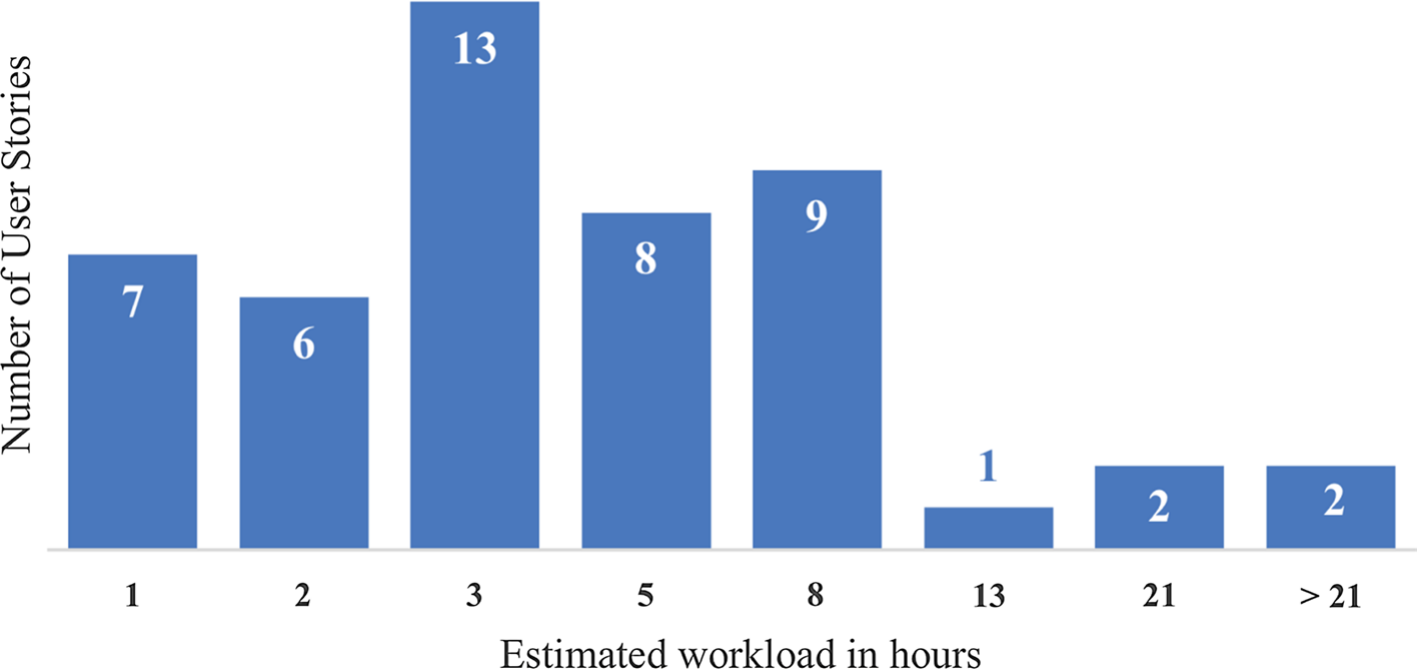
Effort estimation for the not-already-implemented ideas for $\textit{Tournify}$

We did not assess the usefulness of the ideas because of the product stage: at the time of our study, $\textit{Tournify}$ was a very recent product and the company had to balance the inputs with their own growth strategy. We did, however, ask the lead developer to estimate the effort required for implementing the ideas using the Fibonacci sequence (1, 2, 3, 5, 8, 13, 21), with one story point corresponding to one hour of work. Nine ideas were not estimated, since seven referred to features that were already implemented but overlooked by the requester, while two could not be estimated because of their vagueness. 90% (43/48) of the estimated ideas can be developed within one workday, according to this estimation (see Fig. 4).

The $\textit{Tournify}$ case leads to two major findings. First, the large number of user stories with relatively little effort (see Fig. 4) suggests that it is viable to expect specific features that can be easily implemented. Second, the users of the app expressed general appreciation for this way of being involved in the evolution of the product, both through their questionnaire and via follow-up comments such as “every user gets new ideas while using $\textit{Tournify}$ on their tournament” and “a fantastic way to improve the application”.

### S-Sys

As explained in Sect. 5.1, *S-Sys* served to assess whether the deployment of the *CREUS* method would deliver ideas that are comparable to those elicited via established techniques and whether they could lead to additional, previously unidentified ideas. The results were measured in terms of (i) user engagement, (ii) user origin, (iii) appreciation of *CREUS*, and (iv) quality and usefulness of the ideas. The full results are presented in our previous work [13]. Here, we only offer some highlights on user origin and on the usefulness of the ideas.

The statistics in Table 3 show that, in the *S-Sys* study, *CREUS* allowed to reach one of the main goals of the case study: over 58% of the total number of participants were operational employees. On the positive side, this shows that operational employees—a user category that would seldom be included using established elicitation methods—were reached and that they delivered substantial input. Yet, middle management was the most active group on the platform with a higher number of ideas per user. This can be expected as military culture is structured around rank and people with higher rank are more likely to participate in strategic discussions.

**Table 3:** Activity per user type in the *S-Sys* case study (N=135)

Origin	% of total	Per user activity		
		Ideas	Votes	Logins
Operational employee	58.52%	0.23	2.66	1.84
Middle management	8.15%	0.82	3.18	2.55
Non-targeted employee	33.34%	0.11	0.77	1.55

To check the usefulness of the ideas, the two requirements engineers of *S-Sys* (who also did the earlier RE work for *S-Sys* using established elicitation methods) judged all ideas based on the KANO model [49], and determined whether the idea was gathered earlier. The results are given in Table 4. Two of the 32 ideas were unrelated to the goal of the elicitation and are therefore excluded from further analysis. 19 of these 30 ideas were identified in an earlier stage, five were partly identified in an earlier stage, and six were completely new.

**Table 4:** Usefulness of the ideas in the *S-Sys* case study, assessed by the two analysts who conducted the elicitation without *CREUS*

Measurement	Value	# Ideas
KANO model	Must-be	13
	One-dimensional	10
	Attractive	7
Gathered earlier	Completely	19
	Partly	6
	Not at all	5
Complete for dev teams	Yes	11
	No	19

When evaluating the ideas according to the KANO model, 13 of them were must-be requirements, 10 were one-dimensional (i.e., detrimental if not implemented, useful when implemented), and seven were attractive qualities (delighters). If we only look at the ideas which were gathered partly or not at all in an earlier stage, five of these 11 ideas were delighters, two of them were one-dimensional requirements and four of them were must-be requirements. This shows that the CrowdRE activities could contribute to enriching the requirements, although many of the inputs were already identified earlier. Finally, two thirds of the ideas (19/30) were missing important details prior to their use for development: this is not surprising, since involving the crowd of users amounts to allowing people with no RE experience to participate.

The *S-Sys* case study shows that *CREUS* can be used to collect ideas for IT products and that the results are comparable with requirements collected using more established requirement elicitation techniques. Some of the identified ideas were new and not identified in the prior elicitation activities, as CrowdRE taps into a large user base that might otherwise be overlooked. The *S-Sys* case study also confirms that *CREUS* cannot replace other RE efforts, as many requirements collected earlier are not identified using *CREUS*.

### V-Sys

The *V-Sys* case study focused on scaling up *CREUS* to the size of a governmental institution. The results were measured over the same four dimensions as in the *S-Sys* case study: user engagement, user origin, appreciation of *CREUS*, and the usefulness of the ideas. We briefly report on user engagement, user origin, and the usefulness of the ideas here, while a full analysis is in [13].

**Fig. 5:**
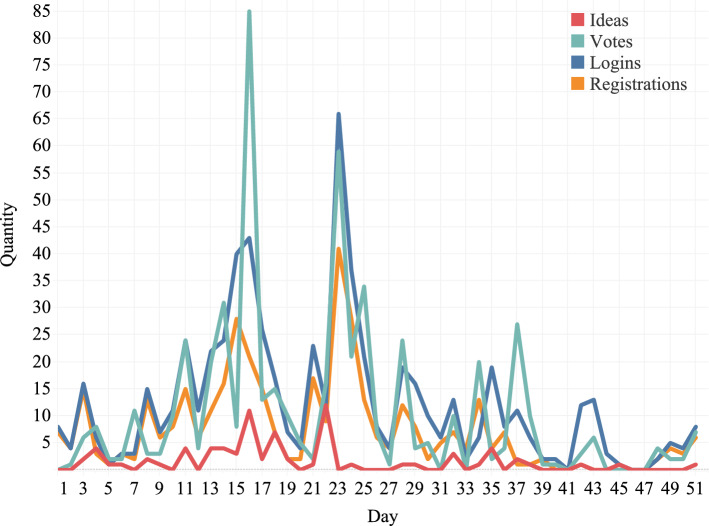
Usage indicators for the *V-Sys* case study plotted over time

Figure 5 summarizes user engagement for the *V-Sys* case. While two peaks existed in the *S-Sys* case (see Fig. 4 in [13]), employees were invited more gradually in the *V-Sys* case, once their brigade commander gave consent. Because of this, the activity on the KMar Crowd platform was more spread out over time (with peaks shortly after a brigade was invited to participate). In total, 385 participants used the platform, 15.8% of the total invited employees. This is a bit lower than in the *S-Sys* case (28.25%) since the larger scale of this case study made it harder for the researcher to pay attention to and to stimulate the participation of all brigades. A relationship between registrations and the other activities is visible, which also occurred in the *S-Sys* case study.

Since the V-Sys case study was executed across different brigades, no distinction is possible between operational employees, middle management and upper management, as each brigade is structured differently. Therefore, we made an analysis based on military rank. 79% of the participants were part of the group targeted, which further strengthens the conclusion on the viability of collecting ideas from groups normally less involved in this process.

For *V-Sys*, no earlier requirements elicitation work was conducted. Therefore, we could not reuse all the same measurements we employed for *S-Sys*. While we kept the KANO model classification, we introduced new measurements. Four requirements engineers (all KMar employees with a role in developing plans for the new system, but not involved in the *CREUS* case study until all the data was gathered), were asked to judge whether the collected ideas would be sufficiently detailed for a minimum viable product (MVP) as well as for a complete and correct implementation of the requirement. The difference between these two can best be explained by whether a requirement is fulfilled completely: in a MVP, the implementation may still be incomplete (e.g., some business rules are not correctly or fully implemented), while in the final product, the requirement should be implemented completely and to the satisfaction of the end-user. Finally, the requirements engineers were also asked whether the idea could be classified as a user story (indicating a single feature), or as an epic (denoting several features). Table 5 summarizes the results.

**Table 5:** V-Sys: usefulness of the ideas, assessed by a pool of analysts

Measurement	Value	Ideas	
		#	%
KANO model	Must-be	40	50.6
	One-dimensional	29	36.7
	Attractive	10	12.7
Enough for MVP		47	59.5
Enough for product		22	27.8
Granularity	Epic	32	40.5
	User story	43	54.4
	Not applicable	4	5.1

Of the 85 gathered ideas (some inputs were split as they contained multiple ideas), six ideas were dismissed, mostly because their implementation would be unfeasible due to legal reasons. Of the remaining 79 ideas, 59.5% were specific enough to implement in an MVP. Only 27.8% of the ideas were specific enough to implement in the final product. The results are, however, promising, as the ideas come from people with no expertise in RE. For granularity, 40.5% of the ideas were classified as epics, 54.4% as user stories. Out of the 5.1% of the ideas that cannot be classified, one regarded stakeholder identification.

The *V-Sys* case study showed that the scale-up of *CREUS* can be done successfully as long as enough energy is spent by the core team to form the crowd. It also showed that over half of the ideas are useful to be implemented in a MVP, but that most ideas need further refinement to be actually implemented in a product. The input of *CREUS* can be seen as a starting point, to get a first grasp of the domain, to identify quick wins (the ‘simple’ ideas sent in) and to identify potential subject matter experts for more complex ideas.

## Qualitative analysis of the elicited ideas (RQ2)

In the second research phase, we address RQ2 by conducting a qualitative analysis of the ideas that were collected through *CREUS* via the deployed feedback platforms in the three case studies. We study the *artifacts* that crowd-based elicitation produces, so to evaluate the quality of these ideas when considered as user requirements. We aim at providing empirical evidence for researchers and practitioners on the quality of the collected ideas.

We analyze a total of 358 ideas: in Table 2, the 248 ideas from $\textit{Tournify}$ $^*$, the 78 ideas from *V-Sys*, and the 32 ideas from *S-Sys*. We exclude some *S-Sys* and *V-Sys* ideas for confidentiality reasons, and a few inputs from $\textit{Tournify}$ $^*$ because clearly not representing a requirement. This leaves us with 341 ideas: 245 from $\textit{Tournify}$ $^*$, 67 from *V-Sys*, and 29 from *S-Sys*. All the ideas from *S-Sys* and *V-Sys* were written in Dutch. For $\textit{Tournify}$ $^*$, 212 ideas were in Dutch, while 33 were in English, as the company expanded their market after the time period when the first research phase was conducted.

All ideas were qualitatively analyzed on the aspects listed in Sect. 3 via the metrics of Table 1. We made the following operationalization choices:

- * User story quality and quality requirements*. Two authors tagged independently the ideas by analyzing both aspects, and they held sessions to reach consensus. For *S-Sys* and *V-Sys*, for confidentiality reasons, we organized physical meetings using printed copies of the ideas. After tagging each idea, the authors compared and discussed their tagging in order to reach agreement. For $\textit{Tournify}$ $^*$, the tagging was conducted in different locations, and the authors held two sessions for reaching agreement.
- *Vagueness*. We first used a Python script (in our online appendix^2^) that searches for the list of words from QUARS++ in a text. Since this list of words is in English, we first translated all the ideas by invoking Google Translate. The returned hits were processed manually by one researcher-author to identify whether the hit was a real occurrence of vagueness.
- *Text readability*. The readability of the requirements was analyzed automatically using the same script for vagueness, by using the textstat 0.7.2 Python library, which calculates the ARI and the Flesch score.
- *Generality vs. Specificity*. For the *S-Sys* and *V-Sys* ideas, items were judged as specific or general by the author-employee, based on whether the idea was specific to one brigade or could be generalized. For the $\textit{Tournify}$ $^*$ items, one researcher judged whether the ideas were related to a single sport or pertained to multiple sports.

For the KMar case studies, due to the format we used to gather ideas, the two fields ‘what would you like?’ and ‘why do you want this?’ were combined to create a user story, representing the ‘I want’ and the ‘so that’ part, respectively. In the examples below, we denote the concatenation of the two fields using a ‘/’ symbol. The role was identified via a separate field; unless necessary, we do not list it here, as its meaning is domain specific. Also, domain-specific terms are substituted by a more general term, typeset in angle brackets: $\langle \ \ldots \ \rangle$.

Each idea has been given a unique identifier which consists of a letter (S for *S-Sys*, V for *V-Sys*, and T for $\textit{Tournify}$ $^*$) and a progressive number. The non-confidential ideas used in this study are available in our online appendix.

### Quality based on the QUS framework

The analysis using the QUS framework aims to assess whether the ideas suffer from the common defects of user story requirements. Within the 13 criteria of QUS [8], we select four that are suitable for user-generated ideas: (i) *well formed*: are both the $\langle$role$\rangle$ and the $\langle$action$\rangle$ specified? (ii) *atomic* : does an idea include a single requirement?; (iii) *conceptually sound*: does the action include the desired feature, and does the reason explain the rationale?; and (iv) *problem-oriented*: is the user story expressed in problem-space terms, or does it indicate a specific solution? While the ideas from the $\textit{Tournify}$ case study period had been assessed with the QUS framework before [12], we analyzed all the ideas in the $\textit{Tournify}$ $^*$ super-set with two taggers, in order to offer more reliable results. The results are shown in Table 6. In addition to the number of defects and the percentage of user stories that exhibit that defect, we present a count based on the number of violations per user story.

**Table 6:** Violations of criteria from the Quality User Story (QUS) framework

Quality violations	S-Sys		V-Sys		Tournify$^*$	
	#	%	#	%	#	%
Not well-formed	7	24.1	12	17.9	0	0.0
Not atomic	10	34.5	18	26.9	33	13.5
Not conceptually sound	3	10.3	3	4.5	11	4.5
Not problem-oriented	7	24.1	23	34.3	46	18.8
Ideas with:						
No violations	12	41.4	26	38.8	164	66.9
One violation	10	34.5	26	38.8	71	29.0
Two violations	6	20.7	12	17.9	9	3.7
Three violations	1	3.4	3	4.5	1	0.4
Total ideas	29		67		245	

The $\textit{Tournify}$ $^*$ data set contained more ideas with no violations: 66.9% vs. circa 40% for the other cases. One likely explanation is the wizard-like template (Fig. 2), which fosters users to express short ideas that stick to the template. Another reason is that $\textit{Tournify}$ $^*$ ideas were gathered for improving an app, rather than for replacing a legacy information system: ideas that indicated bugs to fix or functionality to be improved, which are prone to violating problem-oriented or conceptually sound, were less common for $\textit{Tournify}$ $^*$.

Across all three case studies, the most violated criterion was problem-oriented. As the ideas were posted by users in general and not by requirements engineers, this can be expected, as users are not familiar with the importance of problem-orientation in RE. For example, see idea 40 for *V-Sys*:

**Table:** 

**V-40 ***Change the layout to indicate which steps you need to take. Activating or deactivating a step is so hard to see that sometimes the wrong steps are deactivated. / To prevent that, recovery processes need to be made.*	

The employee who submitted this idea still thinks about manually activating or deactivating a step to progress in the workflow, while the new system might automatically determine the next steps based on a workflow engine. This shows that the employees sometimes find it hard to think of their needs outside the context of the current system. This can also be seen in idea V-62:

**Table:** 

**V-62** *Create a menu in* $\langle$system$\rangle$ *where an employee can edit their rank and workplace to the correct data. The menu should only be accessible for the employee with perhaps an approval of this information by an approver. / This relieves the helpdesk of unnecessary work, which can be edited by the employee him/herself. This ensures the information in formal reports are correct and don’t need to be changed using eventually an additional formal report.*	

The employee is discussing a way to specify their role in the system, while this might also be done automatically using single sign-on. V-62 also violates the atomic criterion, as it discusses both (i) the possibility of letting staff change their own information, and (ii) an approval system that lets the manager of that employee check the information.

We encountered several cases in which multiple requirements were expressed in the same idea, also in the $\textit{Tournify}$ $^*$ case. For example, T-14 discusses both the option to show video replays and the possibility to show a live indicator, which are clearly two different features.

**Table:** 

**T-14** *As an organizer, I want to show the video replays of matches that have happened next to the results with a little icon. I want to also show the LIVE button for games currently in progress, so that I can get more people to watch the game and make the games valuable.*	

The well-formed quality was never violated for $\textit{Tournify}$ $^*$, most certainly thanks to the wizard of Fig. 2, while violations occurred for the KMar case studies. An example of a violation comes from *S-Sys*:

**Table:** 

**S-26** *[As a team lead, I want to] Improve the registration of goods and make this more simple. [so that I can] Make sure a connection exists between* $\langle$new system$\rangle$ *and * $\langle$other system$\rangle$. *Prevent double entry of goods, make this process easier. Make sure there is a better overview of where goods are at a certain time.*	

Although the violation is not immediately clear, the participant who entered this idea indicated an incorrect role (‘team lead’, while it should be ‘operational employee’) in the third field on the form. Therefore, the well-formed condition was violated, as the idea did not contain a (correct) role.

Conceptually sound violations did not occur often and were mostly due to the inadequate rationale given in the $\langle$benefit$\rangle$: often, the feature was repeated as the rationale. An example of this is idea T-168:

**Table:** 

**T-168 ***As an organizer, I want to keep a top scorer list, so that I can collect top scores.*	

Ideas with three violations were rare: in total, four ideas. One idea which violated three qualities is T-21:

**Table:** 

**T-21 ***As an organizer, I would like 1) the possibility to assign referees to an event, 2) push notifications: is it possible to write/generate a push notification ourselves with a message, so that we can reach individual teams during a tournament.*	

This idea is not atomic (as it contains two separate requirements), it is not conceptually sound (the rationale of the first part is not explained), and it is also not problem-oriented (the second part proposes a specific solution)

202 ideas did not include any violation. For example:

**Table:** 

**S-03 ***Search functionality which let you search on call sign so you can see all incidents you need to enter data in one overview. / Often it is searching to find all incidents in* $\langle$old system$\rangle$ *of which you need to enter data for.*	

Ideas with no violations were generally short, as one would expect for a user story. They did not refer to the legacy systems, and they did discuss the process that the user wanted to support with the (new) system.

### Quality requirements based on ISO/IEC 25010

We used five qualities from the ISO/IEC 25010 standard, which were found to be among the most common in user reviews [50], to determine whether the user stories could be associated with specific quality aspects, in addition to functional concerns. The quantitative results are presented in Table 7.

**Table 7:** Analysis of whether the ideas would pertain to quality requirements

Property	S-Sys		V-Sys		Tournify	
	#	%	#	%	#	%
Reliability	0	0.0	3	4.5	1	0.4
Performance	0	0.0	1	1.5	0	0.0
Security	0	0.0	1	1.5	3	1.2
Compatibility	14	48.3	23	34.3	17	6.9
Usability	12	41.4	25	37.3	55	22.4
Ideas with						
No properties	5	17.2	20	29.9	169	69.0
One property	22	75.9	42	62.7	76	31.0
Two or three properties	2	6.9	5	7.4	0	0.0
Total ideas	29		67		245	

The KMar ideas from *S-Sys* and *V-Sys* contained, in percentage, more user stories that could be associated with software qualities than the $\textit{Tournify}$ $^*$ ones. 69% of the $\textit{Tournify}$ $^*$ ideas could not be linked to qualities, while this percentage is 29.9% for *V-Sys* and 17.2% for *S-Sys*. A possible explanation is the different domain: while *S-Sys* and *V-Sys* are information systems that enable well-defined business processes, the $\textit{Tournify}$ app can be extended to support additional sports, hobbies (e.g., cards), or e-sports.

The most common property in the KMar ideas is compatibility. It might be explained by the nature of the considered information systems, which need to communicate with those of other governmental institutions. Indeed, many ideas focused on improved interoperability (a sub-aspect of compatibility in ISO/IEC 25010 [46]) between the KMar and other institutions. We cannot share these ideas because they are classified. However, we share an example about linking the systems with APIs to make them more robust and precise:

**Table:** 

**V-45 ***When you do dynamic patrols and want to report on these, this is not possible for certain locations because they don’t exist. My idea is to connect a street book to* $\langle$old system$\rangle$, *so we can report on all our dynamic patrols. / To make our work easier and to create a complete picture of what we do and where.*	

The users of $\textit{Tournify}$ also expressed compatibility-related ideas, such as T-47, which specifically mentions a push notification service outside of the app:

**Table:** 

**T-47 ***As an organizer, I want to send notifications to a participant. Is it possible to integrate Pushbird in one way or another, so that I can improve the involvement of the participant and can actively send him/her information?*	

After compatibility, usability was mentioned the most in the collected ideas. Most of the time, usability was not mentioned explicitly but ideas were written with the clear intent of improving the usability of the system.

**Table:** 

**S-11** *A tab functionality just as the tabs in your internet browser. This because then it would be possible to have multiple entries open at once. / This saves actions and thus time.*	

The KMar case studies focused on information systems that supported users in their work duties. This might explain why the usability property was more common in the KMar ideas than in the $\textit{Tournify}$ $^*$ ones: the participants of the KMar study are required to use the system—and benefit directly by its usability—, while the $\textit{Tournify}$ users could easily switch to a competing app. The ideas which described usability within $\textit{Tournify}$ $^*$ focused on making operations easier in the system, often expressing small changes that would make common operations within the system easier to perform:

**Table:** 

**T-134 ***As an organizer, I want the possibility to copy the division’s structure within one tournament, so that I set up of the local tournament can be done more quickly.*	

Very few ideas could be linked with Reliability, Security and Performance, probably because the users do not think of these qualities that are somehow ‘invisible’ [50]. This is another confirmation that crowd-based elicitation is not a complete replacement of established elicitation methods.

An example of a security-relevant idea is V-62, which was listed in Sect. 6.1. It shows that participants think about manually changing their security role without waiting for the help desk, which takes time. It does, however, also show that this participant only thought of the security of the system as the current implementation hampers their productivity. For $\textit{Tournify}$ $^*$, some ideas were clearly about security, such as T-241, which is shown in Sect. 6.5. T-87 is another example:

**Table:** 

**T-87 ***As an organizer, I want to limit an account so I can share it, so that I can organize a tournament with multiple people but do not have to give out admin-rights to everyone.*	

Idea V-22 shows an idea that relates to reliability: this arises from a bug in the current system, which is unlikely to exist in the new one:

**Table:** 

**V-22 ***In the* $\langle$old system$\rangle$, *if you enter data and press the enter button, you will lose the information entered (except when you clicked on ‘save’). Maybe this ‘handy’ function can be removed, and a function that automatically saves information can be added. / User-friendliness, no loss of data or re-entering information again.*	

Ideas that could be associated with two or more quality properties were rare and did not occur at all in the $\textit{Tournify}$ $^*$ case. For KMar, most were combinations of usability and compatibility, for instance V-72, showing how compatibility improves efficiency and, consequently, higher user-friendliness.

**Table:** 

**V-72 ***Connecting* $\langle$Schiphol Airport system$\rangle$ *to * $\langle$old system$\rangle$. *To make it more easy to add flights to a process / Saves time.*	

### Specificity vs. generality

Table 8 presents the results of the analysis of the specificity of the ideas. Across all three case studies, general ideas (not pertaining to a specific user type or usage context) were the most common. The KMar case studies did contain slightly more specific ideas, but overall the studies do not differ much.

**Table 8:** Specific versus general ideas

Item	S-Sys		V-Sys		Tournify	
	#	%	#	%	#	%
Specific	4	13.8	12	17.9	25	10.2
General	25	86.2	55	82.1	220	89.8
Total ideas	29		67		245	

For the KMar case studies, specific ideas were mostly about niche tasks to be performed in the systems. Employees indicated, sometimes even inside the user story, that such functionality should not be overlooked. For example, see V-72, presented in Sect. 6.2: a link with the Schiphol Airport system only benefits the operational brigade which performs its duties at that airport. For $\textit{Tournify}$ $^*$, specific ideas were those mentioning, or applicable only to, a single sport. An example is idea T-152, which asks explicit support for hexathlons.

**Table:** 

**T-152 ***As an organizer, I want to be able to use Tournify for hexathlon, so that I can use Tournify for different events.*	

Most ideas, however, were general; many are presented in the paper, such as those about user-friendliness, which are applicable for the whole application, not only a specific user type. This shows that the participants in these case studies are acting ‘as a crowd’: if most ideas were specific, the interest of the crowd as a whole could be overlooked, leading to many ideas that are shared by only a few members [20]. This crowd behavior can also be seen by comparing the average number of votes according to the two categories: Table 9 shows that general ideas typically received more votes from the crowd than specific ones.

**Table 9:** Average number of votes per category

Average # of votes	S-Sys	V-Sys	Tournify
Specific	11.25	2.85	1.24
General	5.44	4.65	2.15

A notable exception is *S-Sys*, where the average number of specific votes was considerably higher. This might be due to the low number of specific ideas (only four), which attracted a high number of votes.

Although a high number of votes in the general category shows that the crowd is able to prioritize the group interest over that of individual interests, specific ideas are needed to ensure that niche requirements are considered for inclusion in the system. These ideas may be overlooked if the core team only considers the vote count when prioritizing the ideas to implement. In that case, the niche participants would need to mobilize everyone in their group to vote on their specific idea (as it happened in the *S-Sys* case study). The fair treatment of minorities strengthens the importance of having a core team that analyzes all inputs. Another approach would be to apply CrowdRE with a sub-crowd that consists only of people in the niche group. The trade-off between general and specific ideas is subject to further research.

### Readability

**Fig. 6:**
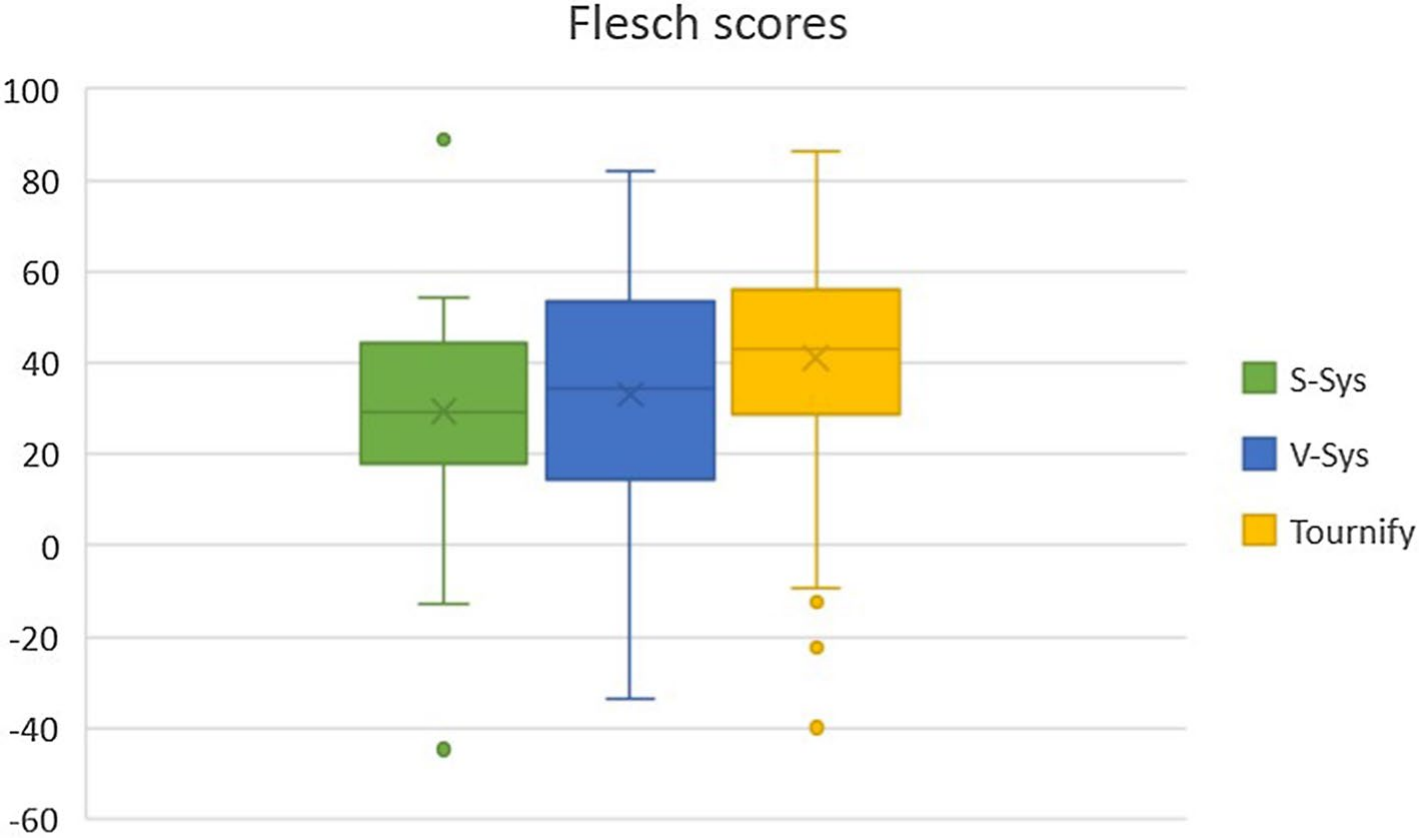
Boxplot of the Flesch-scores for *S-Sys*, *V-Sys* and $\textit{Tournify}$ $^*$

**Fig. 7:**
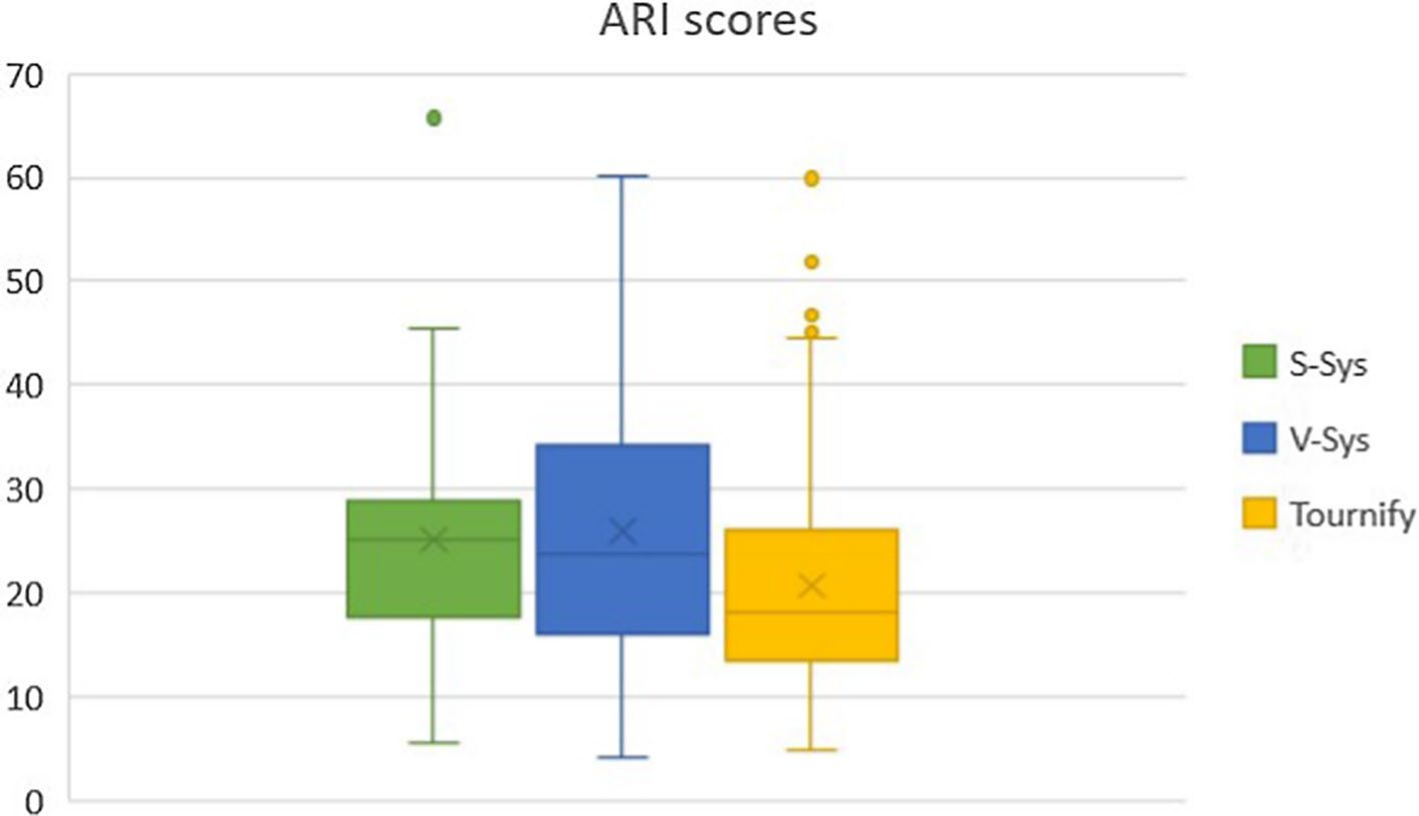
Boxplot of the ARI-scores for *S-Sys*, *V-Sys* and $\textit{Tournify}$ $^*$

We assessed the readability of the ideas as general text, via automated readability scores, to identify differences in the estimated readability of the ideas across the cases. We selected the Flesch-score and ARI because of their popularity and as they rely on slightly different way of estimating complexity (see Table 1). The results are shown in Fig. 6 and in Fig. 7. If a text is harder to read, the Flesch-score is low, and the ARI score is high.

Of the three case studies, the $\textit{Tournify}$ $^*$ ideas were the most readable according to the indices (Flesch $\overline{x}$ = 41.11, ARI $\overline{x}$ = 20.80). The spread of those scores was higher than the scores of *S-Sys*, but the number of data points for *S-Sys* was also significantly lower (29) than the number of data points for $\textit{Tournify}$ $^*$ (245). The readability for *S-Sys* (Flesch = 29.48, ARI = 25.12) and *V-Sys* (Flesch = 33.02, ARI = 25.99) is lower according to the Flesch and ARI scores. The most likely explanation is the $\textit{Tournify}$ wizard, which prompted the users for concise user story parts that would lead to a single-sentence user story. Other possible causes are the automatic translation necessary to perform the calculations, and the more specific domain for which ideas were gathered.

The ideas posted for *S-Sys* and *V-Sys* contained more text (on average, 349 characters for *S-Sys*, 421 for *V-Sys*, and 264 for $\textit{Tournify}$ $^*$), which might also have led to a less favorable readability score. In general, all three cases had readability scores that indicated very complex texts. This might be because the indices used are mostly meant to be applied to larger texts, such as books, and the algorithms may not perform as expected on short text. Experimenting with other algorithms that could be used to determine the complexity of crowd-generated ideas is subject to further research.

Although the ideas were hard to read according to these scores, it does not mean they were unusable. First, the analysts may just need more time in order to fully comprehend the ideas. Second, the algorithms that estimate readability do not possess the domain knowledge that analysts and stakeholders have.

### Vagueness

The vagueness of the collected ideas was assessed using the method discussed in Sect. 3. We identified the occurrence of each of the vague words from QUARS++ [43], then calculated *vagueness hits* by counting the number of vague words per idea. Then, we manually processed the hits to determine whether they would represent a real instance of vagueness or not and, if not, why. The distribution of the hits per idea is presented in Table 10.

**Table 10:** Distribution of vagueness hits

# Hits per idea	S-Sys		V-Sys		Tournify	
	#	%	#	%	#	%
None	8	27.6	25	37.3	153	62.5
One	11	37.9	21	31.3	72	29.4
Two	6	20.7	9	13.4	15	6.1
Three	3	10.3	5	7.5	5	2.0
Four or more	1	3.5	7	10.5	0	0.0
Total ideas	29		67		245	

The *S-Sys* and *V-Sys* ideas contained more vagueness hits than $\textit{Tournify}$ $^*$ when using the QUARS++ word list. Most of the $\textit{Tournify}$ $^*$ stories did not contain vague words (62.5%), and the others had only one or two vague words. The *S-Sys* and *V-Sys* ideas contained more vague words: only 27.6% of *S-Sys* and 37.3% of *V-Sys* ideas did not contain vague words. This discrepancy may be due to the higher number of words in the ideas from the KMar case studies.

Since most of the $\textit{Tournify}$ $^*$ ideas and all the KMar ideas were written in Dutch, while the list of words from QUARS++ is in English, the automatic translation of the ideas might have affected the results. Therefore, we further analyzed the results by classifying them based on the type of hit gathered: true positive of vagueness, false positive because the vague word gets clarified in the sentence, false positive because the vague word is used in a common phrasal expression, false positive because of a typo, or false positive because the vague word is a domain term. The results of this analysis are presented in Table 11.

**Table 11:** Quantitative results of vagueness analysis

Statistic	S-Sys		V-Sys		Tournify	
	#	%	#	%	#	%
True positive	10	27.0	27	31.0	29	24.8
False positive						
Clarified by sentence	4	10.8	20	23.0	42	35.9
Phrasal expression	22	59.5	40	46.0	40	34.2
Typo	0	0.0	0	0.0	1	0.8
Domain term	1	2.7	0	0.0	5	4.3
Vagueness hits	37		87		117	

Overall, over 70% of the hits were false positives. Most of the false positives were words that were in the vagueness list but were used in the ideas as a phrasal expression. An example of this is idea S-29, which contained the phrasal expression ‘makes it possible to’ (*possible* is the vague word here):^3^

**Table:** 

**S-29 ***Dashboard Insert function in the module where everyone can make a management dashboard yourself to keep an eye on his own processes. Depending on the function of the employee, various overviews are added to the dashboard. / Makes it possible to better steer on their own work and work of colleagues.*	

Another example of a phrasal expression that led to a false positive is idea T-241, in which the word ‘nice’ triggered the vagueness algorithm, even though the phrasal expression ‘nice (to have)’ is not vague:

**Table:** 

**T-241 ***As an organizer, I want the assurance that an e-mail address that is passed on when registration is really valid, so that a confirmation also knows. For this you could use a third party check, such as https://www.milgun.com/email-verification-service, so that we have more certainty about the mail. Can see that an email is open is also nice. You probably use an email distribution API and you can simply show that metrics (sent / open) in the GUI to the user.*	

Some vague words were clarified elsewhere in the sentence, and therefore should not be seen as vague. In idea V-28, the word ‘user-friendly’ is flagged as vague, but the context of the sentence clarifies this term:

**Table:** 

**V-28 ***In the* $\langle$old system$\rangle$ *there are pages that you have to press saving, but at the same time also goes further. If you press further, you will lose everything from the page, this should be more user-friendly with a popup notification (you are sure that you do not want to save this page) or just only show one option with further (automatically save). / Usability*	

Some hits denoted a truly vague idea, for which we could not determine what the participant meant with certain words. An example of this is idea V-06, where the words ‘legible’ and ‘user-friendly’ were flagged as vague:

**Table:** 

**V-06 ***Now it is true that you have to enter a stranger in the* $\langle$old system$\rangle$ *and again in the* $\langle$KMar system$\rangle$, *for the future if it is possible to link these systems. Also make user-friendly*. *In the* $\langle$old system$\rangle$ *a number of option are not legible which must be checked. The processes are also not clearly defined. Where there must be a cross then the rest which does not apply. This is clearer. Also save a screen before continuing. Now you can continue without saving, with the result that people have lost everything. Making more user-friendly, if the Enter key is accidentally touched that does not hits the entire system on tilt. By linking data does not always have to be filled in the same data, in other words, time saving. / The above makes it all more user-friendly and easier to process.*	

In this case, the first hit with ‘make user-friendly’ is vague: it is not clear what the user sees as ‘user-friendly’. The same applies to ‘legible’: even with basic domain knowledge this term is too vague and hinders the idea to be implemented as-is. Some $\textit{Tournify}$ $^*$ ideas also contained truly vague words, such as idea T-113. It is not clear what is meant with a ‘large’ screen and implementation choices might depend on how large the screen is going to be.

**Table:** 

**T-113 ***As an organizer, I want to show the top score in the slide, so that we can immediately show this on a large screen*	

Although the vagueness list might help to quickly identify ideas that need further refinement, a true positive rate of 27.6% over 241 hits shows that a basic lexical approach is not sufficient for crowd-generated ideas. Possible ways to overcome this challenge is to automatically split multiple ideas in the same text, thus shortening the text, or having language-specific lists of vague words, which could increase accuracy. Once the TP rate for vagueness is sufficiently high, an automated system may be introduced to support *CREUS* by alerting a participant of vague words while s/he is sending in an idea, prompting her/him to refine the idea by avoiding or clarifying vague terms.

## Key findings and conclusions

We first present the key findings we could identify *empirically* from the two phases of our research, and discuss how these relate to existing literature. Then, we explicitly address the research questions RQ1 and RQ2.

- *KF1. In addition to their functional orientation, many of the crowd-generated user stories can be associated with quality aspects.*
  Virtually all of the posted ideas include functional aspects. This is likely explainable by the user story notation (“As a ... I want”), which highlights the interaction between the user and the system and prompts the user to specify some expected functionality. This is also pointed out by Cohn’s popular book on user stories [14, p. 4]: “A user story describes functionality that will be valuable to either a user or purchaser of a system or software”. Besides this functional orientation, a good number of user stories (83% for *S-Sys*, 70% for *V-Sys*, and 31% for $\textit{Tournify}$ $^*$, see Table 7) can be associated with quality aspects. While some of these links are implicit (T-87 refers to security by indirect words such as ‘password’), others are explicit: the user who wrote T-113 (Sect. 6.5) explains how showing the top score in the slide would contribute to user friendliness by making the score immediately shown on a large screen. Our finding aligns with the work by Gilson [51], which showed how quality aspects can be found in one in four user stories from a publicly available collection [52].
- *KF2. Crowd-generated user stories can be associated mostly with two software qualities: usability and compatibility.*
  In our three cases, usability is the quality aspect that user stories can more consistently be associated with, having a varying percentage from 22% ($\textit{Tournify}$ $^*$) to 41% (*S-Sys*). Compatibility is also highly mentioned, especially in the *S-Sys* and *V-Sys* cases where the information systems under design are highly linked to other systems. This finding aligns with the study by Groen et al. [50] on app store reviews, which showed how usability is the most prominent quality aspect, while other qualities are ‘invisible’ to the users [50]. The prevalence of these two software qualities may vary with other types of software systems. However, previous studies show that usability is a prominent quality aspect that can be often found in user reviews [50] as well as in documented requirements [53], and that it is considered of high importance [54]. On the other hand, it is plausible that the prevalence of compatibility in our cases has to do with the type of systems. The coverage of quality aspects could increase should the crowd members be more aware of RE basics. However, our goal was to study how lay, untrained users would contribute.
- *KF3. The elicited ideas are proto-requirements, but further refinement is needed.*
  Circa 40% of the ideas that we analyzed in Sect. 6 include at least one quality violation according to the QUS framework [8], showing that the ideas elicited via *CREUS* do not always represent a ready-to-use requirement. In particular, the most common violations concern expressing multiple requirements in the same idea (non-atomic) and writing a solution rather than the problem to be solved (solution-oriented). This is also confirmed by the analysis of usefulness for *S-Sys* (Table 4) and *V-Sys* (Table 5), which show that only 36.7 and 27.8% of the user stories are complete enough for development teams. Thus, *CREUS*’ inputs need to be further analyzed by requirements engineers. This is in line with our expectations, as we see *CREUS* as an additional elicitation technique that complements existing processes, rather than a replacement.
- *KF4. Steering the crowd is essential for sustained interaction.*
  The activities of monitoring the crowd, responding to ideas, and writing summaries are necessary to foster and to retain participation. For *S-Sys* and *V-Sys*, we witnessed peaks of engagement (e.g., see Fig. 5) when the core team’s activity was higher. This is also visible for $\textit{Tournify}$: when we compare the density of ideas for the case study period against the post-experimental period, we have 1.62 ideas per day (57 ideas in 35 days for $\textit{Tournify}$ in Table 2) versus 0.2 ideas per day ($248-57=191$ ideas / $1000-35=965$ days for $\textit{Tournify}$ $^*$). Therefore, pull elicitation platforms should be considered only as part of a method in which the requirements engineers are involved to monitor and push the interaction. However, Kolpondinos and Glinz [2] identified a long-tail effect concerning user activity; it is likely that the effect of steering the crowd will gradually vanish over time. The design of effective mechanisms to sustain crowd engagement is still an open topic of research [2, 20]. Extrinsic motivation techniques such as gamification [9] do not always work: in the KMar studies [13], the participants did not perceive game elements as useful.
- *KF5. The crowd-generated ideas are mostly general in nature, with a smaller part focusing on specific user types or usage contexts.*
  Since crowd members are not trained in RE, one possible challenge was that their ideas could pertain only to their specific use case. The results shown in Table 8 and in Table 9 seem to indicate that most of the expressed features are not only general (thus, not specific to a given user type or usage context), but also that these general ideas have on average more votes than the specific ones. One exception is the *S-Sys* case, where the specific ideas received more votes on average, but this is probably due to the low number of specific ideas.
- *KF6. Simple automated techniques for vagueness identification lead to a significant number of false positives for crowd-generated ideas.*
  We applied the lexical technique for vague requirements detection proposed by Ferrari et al. [43] in the context of requirements for safety-critical systems. While they report precision of 45, 56 and 70% in three studies, we see that the results with crowd-generated ideas are much lower: 27% for *S-Sys*, 31% for *V-Sys*, and almost 25% for $\textit{Tournify}$ $^*$. This may have to do with the fact that our user stories are written by users and not by requirements engineers. Therefore, the outputs need to be reviewed by human experts in order to determine true occurrences of vagueness, and further research is necessary to improve the accuracy of vagueness detection techniques.
  Based on the analysis of the key findings and the other materials presented in this paper, we can now provide our answer to the research questions.
- *RQ1. What method can support requirements engineers in the adoption of crowd-based elicitation via pull feedback?*
  Through the conduction of three case studies using canonical action research, we have iteratively devised the *CREUS* method that is presented and discussed in this paper. To provide clear guidance, we formalized the method description using a PDD. *CREUS* complements, rather than replaces, established elicitation techniques. As shown in Sect. 5, users appreciate being involved, and *CREUS* has the potential to deliver some ideas that were not considered earlier. *CREUS* is not prescriptive; while the key activities are important for crowd-based elicitation via pull feedback, their interleaving and duration need to be adapted to the context. One of the pillars of the method is steering the crowd (KF4); yet, the feedback intensity is likely to diminish over time.
- *RQ2. What types of ideas are prevalent when deploying crowd-based elicitation methods via pull feedback?*
  The major novel contribution of this paper is the analysis of the quality of the crowd-generated ideas. We selected five aspects to measure the quality of the ideas: user story quality, vagueness, text readability, quality aspects that can be associated with the ideas, and generality vs. specificity. Our analysis reveals that a good number of user stories can be associated with quality concerns, in addition to expressing a functional perspective (KF1), with a prevalence of usability and compatibility (KF2). The results confirm the *CREUS* cannot replace other elicitation techniques, not only because of the limited focus on quality concerns, but also because most ideas need substantial refinement (KF3). Yet, the participants seem to be able to think beyond their niche use of the systems, as most of the generated ideas were of general nature (KF5).

## Limitations and future work

### Threats to validity

We discuss the limitations of our research by reporting on the validity threats using the four types of validity suggested by Runeson and Höst for case study research [55]: construct, internal, external, and reliability.

#### Construct validity

 This category assesses whether the operational measures actually align with the researchers’ aims and the research questions.

For RQ1, the cross-case comparison in Table 2 relies on uniform metrics to analyze the size of the crowd and of the produced feedback. These indicators were primarily selected for their availability. However, they do not accurately represent a crowd as a whole: the dynamics of a crowd are a more complex, hard-to-measure notion. Also, given the different goals and contexts of the case studies (see Sect. 5.1), we could not always use the same metrics: for example, we could not assess the usefulness of the ideas collected for $\textit{Tournify}$. These are, however, minor threats, as the collected data provided us with sufficiently rich information for us to analyze and compare the cases.

For RQ2, requirements quality is still subject to academic research. We applied two frameworks (the Quality User Story Framework and the ISO/IEC 25010) to evaluate the quality of the ideas. Although this approach revealed interesting differences and commonalities, we cannot claim that we conducted a comprehensive analysis of requirements quality.

#### Internal validity

 This aspect concerns the causal relationships that are identified in the research.

The *CREUS* method described in Sect. 4 is built on top of earlier work in the CrowdRE community, and it is the result of an iterative design process, as explained in Sect. 5: the three case studies adopted different versions of *CREUS*. This threat to RQ1 is mitigated by the fact that we do not draw comparisons on the effectiveness of our method compared to other CrowdRE approaches, besides the quantitative overview in Table 2 that is only meant to show the size of the case studies in comparison to previous research.

The results across our three case studies may be affected by the evolution of the method, although the changes are minor (see Sect. 5.1). The most notable difference is in the platforms, which used different templates for formulating the user stories: the wizard used in $\textit{Tournify}$ prevented, for instance, user stories that are not well formed (RQ2).

To identify causality rather than spurious correlation (RQ2), we analyzed a large number of data items. Despite the 341 analyzed ideas and 325 active users in total, the case studies were diverse in terms of system type and domains. As a mitigation, we made sure that at least one case ($\textit{Tournify}$) considered a different domain than the other two, but even then the results obtained may be affected by the selected domains. In particular, it is possible that the most common software qualities (see finding KF2) may differ in other domains, e.g., reliability is very common for video-games [50]. Additional studies with *CREUS* and analogous methods are needed to draw stronger implications.

#### External validity

 This category concerns the extent to which it is possible to generalize the findings beyond the investigated cases.

While canonical action research enables studying the effect of an intervention within a real-world context, this requires adapting the research method to a specific problem that is faced by the organization. Therefore, the *V-Sys*, *S-Sys*, and $\textit{Tournify}$ cases (RQ1) are not a homogeneous set of cases from which conclusions can be drawn. For example, *V-Sys* and *S-Sys* are similar types of information systems, while $\textit{Tournify}$ focuses on a mobile app. Also, the scale of the crowds is different, both in terms of potential crowd and engaged crowd. Finally, the number and complexity of the collected ideas varies.

To mitigate this inherent challenge, we focused on comparable time frames in Sect. 5 (RQ1), and the qualitative analysis in Sect. 6 (RQ2) takes into account the cases’ characteristics. Certain findings, especially KF2 and KF5, require additional research in order to obtain more general findings.

#### Reliability

 This aspect concerns the impact of the specific researchers on the results.

For the data collection for RQ1, the core teams interpreted the crowd ideas, which could have been mis-interpreted. This risk was partially mitigated by using domain experts in the KMar cases and by selecting a simple domain for the Tournify case, but erroneous interpretation issues might still have occurred.

For RQ2, we let two researchers independently classify ideas. In case of disagreement, a discussion was held until the disagreement was resolved. Table 12a and b shows the inter-rater agreement for the tagging using the Quality User Story framework and the ISO/IEC 25010 qualities, respectively. We report both the percentage of agreement (%) and Cohen’s kappa ($\kappa$). The value n/a applies to (i) $\kappa$ whenever the raters do not have at least one agreement on the positives and one agreement on the negatives; (ii) % whenever there are no true positives at all.

**Table 12:** Inter-rater reliability calculations

Quality	S-Sys		V-Sys		Tournify	
	$\kappa$	%	$\kappa$	%	$\kappa$	%
(a) Quality User Story (QUS) Framework						
Well-formed	0.79	93.1	0.83	95.5	n/a	100.0
Atomic	1.00	100.0	0.72	89.6	0.60	91.4
Conceptually sound	0.33	79.3	0.57	94.0	0.37	95.1
Problem-oriented	0.37	75.9	0.44	73.1	0.35	81.6
(b) ISO/IEC 25010 qualities						
Reliability	n/a	n/a	0.48	97.0	0.00	99.6
Performance	n/a	n/a	1.00	100.0	n/a	100.0
Security	n/a	n/a	1.00	100.0	0.50	99.2
Compatibility	0.73	86.2	0.90	95.5	0.47	94.3
Usability	0.60	79.3	0.59	80.6	0.54	85.7

The $\kappa$ of some qualities are rather low, although none of them is lower than ‘fair agreement’ according to the interpretation guidelines by Landis and Koch [56]. This occurs because the datasets are unbalanced. For $\textit{Tournify}$ $^*$, only three ideas had the ‘security’ quality, and initially, the two researchers disagreed as one of them overlooked this. Therefore, the $\kappa$ is low, even though the percentage of agreement in this category is high. Taking the combined results of these two statistics, we believe that the inter-rater reliability is good enough to base conclusions on, especially in light of the follow-up discussions that resolved the disagreements. Thus, the results behind findings KF1 and KF2 are sufficiently reliable.

The specificity vs. generality decision (leading to KF5) was made by a single researcher, the only one who possessed the necessary domain knowledge for the KMar case studies. To increase consistency across the cases, the same researcher also tagged the $\textit{Tournify}$ $^*$ data. The impact of involving a single researcher for this decision is limited, as determining if an idea is specific is straightforward when using the operationalizations described in Sect. 6.

For the vagueness analysis (leading to KF6) and the readability scores, the data set was split. One researcher handled the $\textit{Tournify}$ $^*$ data set, another researcher handled the KMar outputs. While the readability score relied fully on an automated script, the decision on whether a vague word would be a true positive was made by a single researcher, which differed across the case studies. Due to time constraints and confidentiality reasons, this unfortunately could not be done in another way, and this might have resulted in the introduction of rater bias. To mitigate this, the researchers discussed exemplary cases.

### Future directions

In the first research phase, we assessed the ideas using the Kano model, which combines aspects of novelty with the expected impact on user satisfaction. However, that is not a full metric for novelty, and future work should study *idea novelty* using the frameworks from creativity in RE research [57].

In the $\textit{Tournify}$ case, the use of a wizard to express user stories led to shorter and crisper ideas than those we derived from the KMar cases. Future work should investigate more thoroughly how authoring tools (similar to those that support requirements engineers [58]) can assist crowd participants in the task of expressing high-quality requirements. The wizard can also be extended to more interactive techniques such as requirements bots [59].

Furthermore, it might also be interesting to introduce different voting types. This might enable the core team to better understand the importance of the ideas and let the crowd indicate the importance of ideas for multiple factors such as business importance, feasibility, or other factors.

In the second phase of our research, we used multiple frameworks to measure the quality of the generated ideas. It would be interesting to conduct additional research that uses the employed metrics and other ones to estimate the additional time investment that is required to refine the ideas into requirements that can be assigned to development teams. These indicators may also be employed for comparing different methods for crowd-based elicitation.

Our application of vagueness and readability scores showed that the selected state-of-the-art techniques did not prove to be perfectly suitable for their application to ideas generated via *CREUS*. Future research should study which other techniques could be reliably used to evaluate vagueness and readability. These techniques could then be embedded in an automated assistant that nudges crowd members to improve their ideas before submitting them.

Most of the discussed directions aim at enabling a comprehensive assessment of and the explicit comparison between CrowdRE solutions, which is essential for further advancing CrowdRE research.

## Appendix A Concept and activity tables for the PDD of *CREUS*

See Tables 13 and 14.

**Table 13:** Concept table for the *CREUS* method illustrated in Fig. 1

Concept	Description
CROWDRE GOAL	It defines the main objective of the crowd-based elicitation [9], e.g., improving the UI, identifying new functionalities, etc.
FEEDBACK CHANNEL	The platform through which the crowd members express their feedback, for instance, an idea generation portal.
CROWD MEMBER	A crowd participant who accesses FEEDBACK CHANNEL and may express FEEDBACK.
USER STORY	A concise representation of a requirement that expresses an expected *action* the system should support, the *role* who wants the action, and the *benefit* for the *role* [8, 14].
FEEDBACK	Any input provided by the crowd that addresses the CROWDRE QUESTION via the FEEDBACK CHANNEL: either an IDEA, a VOTE, or a COMMENT.
IDEA	A suggestion or a request for the system that a crowd member expresses as an USER STORY.
VOTE	Expresses the positive or negative support to an IDEA. Can be expressed by crowd members who are not the authors of the USER STORY.
COMMENT	A clarification or explanation of an IDEA provided by a crowd member (either the author or another crowd member).
SUMMARY	A recap of a set of FEEDBACK items that is intended to provide an overview of that FEEDBACK.
RESPONSE	A response to an IDEA given by the core team, to show that the IDEA is being considered. Preferably, RESPONSEs show whether IDEAs are being included in the product under consideration, and show what the argumentation for ex/inclusion is.
TIMELINE	An overview of the temporal horizon of the expected implementation of the FEEDBACK. The level of details depends on the development method.
PRODUCT BACKLOG	The master list of all functionality desired in the product [14], each of which is called a BACKLOG ITEM.
BACKLOG ITEM	A single unit of work that is placed on the PRODUCT BACKLOG [60], which may possibly build on the FEEDBACK provided by the crowd.
SPRINT	A short iteration (2-4 weeks, typically) through which a subset of the PRODUCT BACKLOG (thus, a number of BACKLOG ITEMS) is moved onto a sprint backlog, which is implemented in that iteration.

**Table 14:** Activity table for the *CREUS* method illustrated in Fig. 1

Activity	Sub-activity	Description
CrowdRE preparation	Create core team	The core team oversees and interacts with the crowd after its deployment, in order to support and retain their engagement.
	Define goal for the crowd	The core team defines the CROWDRE GOAL for the crowd to address, so to maximize the relevance of the collected ideas [9].
	Setup feedback channel	A FEEDBACK CHANNEL is selected and configured so that FEEDBACK can be collected from the crowd [1].
	Deploy crowd	The CrowdRE elicitation is kickstarted by opening up the FEEDBACK CHANNEL and by inviting the crowd members to join.
Idea generation	Generate ideas	CROWD MEMBERs formulate IDEAs via the FEEDBACK CHANNEL using the USER STORY format.
	Vote on ideas	CROWD MEMBERs post up- and down-VOTEs on previous IDEAs to express their support [61].
	Discuss ideas	CROWD MEMBERs post COMMENTs that elaborate on the previously posted IDEAs.
	Monitor crowd	The core team oversees the progress of the crowd and sends stimuli when necessary to boost participation.
Refinement	Write summary	The core team puts together a SUMMARY of the FEEDBACK obtained, and shares it with the crowd to give a quick overview.
	Respond to ideas	The core team adds RESPONSEs to the IDEAs to clearly indicate that they are currently being considered
	Generate ideas	As earlier: the generation of IDEAs continues, also based on the RESPONSEs and the SUMMARY.
	Vote on ideas	As earlier: the casting of VOTEs continues
	Discuss ideas	As earlier: more COMMENTs are posted
	Monitor crowd	The overseeing process continues, with a focus on supporting convergence for the current IDEAs.
Response and execution	Respond to remaining ideas	The core team writes RESPONSEs to all the remaining IDEAs.
	Develop and share timeline	The core team prepares a TIMELINE for the implementation of the system, based on the crowd-based elicitation. This TIMELINE is shared with the CROWD MEMBERs.
	Invite to focus group	The most active participants are invited to join a focus group [9] that discusses and prioritizes the BACKLOG ITEMS, which become the PRODUCT BACKLOG.
	Execute timeline in sprints	The TIMELINE is executed iteratively by defining and carrying out SPRINTs, each of which gets assigned a number of BACKLOG ITEMs from the PRODUCT BACKLOG.

## Footnotes

1 While CrowdRE promotes the involvement of *all* stakeholders, most studies, including this, focus mainly on current and prospective *users*.

2 https://doi.org/10.5281/zenodo.6966978

3 While all the ideas presented in the paper are manually translated for the readers’ convenience, the ideas presented in the vagueness section are automatically translated and presented as such, as this translation was used to decide whether the idea was truly vague or that it was a false positive.
